# Influence of growth medium on the species‐specific interactions between algae and bacteria

**DOI:** 10.1111/1758-2229.13321

**Published:** 2024-08-21

**Authors:** Kamile Jonynaite, Arunas Stirke, Henri Gerken, Wolfgang Frey, Christian Gusbeth

**Affiliations:** ^1^ Laboratory of Bioelectrics, Department of Functional Materials and Electronics State Research Institute Center for Physical Sciences and Technology Vilnius Lithuania; ^2^ School of Sustainable Engineering and the Built Environment, Arizona Center for Algae Technology and Innovation Arizona State University Tempe Arizona USA; ^3^ Institute for Pulsed Power and Microwave Technology Karlsruhe Institute of Technology Karlsruhe Germany

## Abstract

In this study, we investigated a species‐specific algal–bacterial co‐culture that has recently attracted worldwide scientific attention as a novel approach to enhancing algal growth rate. We report that the type of interaction between *Chlorella vulgaris* and bacteria of the genus *Delftia* is not solely determined by species specificity. Rather, it is a dynamic process of adaptation to the surrounding conditions, where one or the other microorganism dominates (temporally) depending on the growth conditions, in particular the medium. Under laboratory conditions, we found that *Delftia* sp. had a negative effect on *C. vulgaris* growth when co‐cultured in a TAP medium. However, the co‐culture of algae and bacteria under BG‐11 and BG‐11 + acetic acid resulted in an increase in algal concentration compared to algal cultures without bacteria under the same conditions. Additional chemical analysis revealed that the presence of different carbon (the main organic carbon source—acetic acid in TAP or BG‐11 + acetic acid medium and inorganic carbon source—Na_2_CO_3_ in BG‐11 or BG‐11 + acetic acid medium) and nitrogen (NH_4_Cl in TAP medium and NaNO_3_ in BG‐11 or BG‐11 + acetic acid medium) species in the growth medium was one of the main factors driving the shift in interaction type.

## Graphical abstract

Influence of growth medium on the co‐culture of Chlorella vulgaris microalgae and *Delftia* sp. bacteria. In the TAP medium, co‐culture reduced the concentration of algal cells by 40% compared to the algae grown alone. In BG‐11, the co‐cultured algal concentration increased by 50% and in BG‐AA by 160% compared to the control.

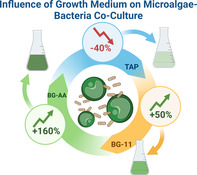

## INTRODUCTION

The importance of microalgae and their valuable co‐products is well established. These microorganisms are a source of bioactive compounds with antibacterial, antiviral, antitumor and immunomodulatory activities (Lu et al., 2021; Raposo et al., 2014; Zhang et al., 2019). Furthermore, microalgae's high growth rate and biomass productivity (the storage capacity can exceed more than half of their dry weight) distinguished them among the biotechnology, food, pharmaceutical and energy industry sectors (Alam et al., 2020). However, to meet the increasing demand for valuable microalgae and their synthesized compounds, optimization of its cultivation process remains a challenge (Rajesh Banu et al., 2020). The most recently suggested alternative to enhance biomass yields and reduce the cost of cultivation is the use of microalgal–bacterial interactions (Krug, Morauf, et al., 2020; Peng et al., 2021). This strategy is particularly advantageous because algae naturally grow in the presence of other microorganisms allowing them to reproduce and thrive even under extreme conditions (Krug, Erlacher, et al., 2020). The interactions are based on the exchange of micronutrients, macronutrients and bioactive compounds provided between organisms (Braga et al., 2016; Cho et al., 2015; Peng et al., 2021). In addition to growth promotion, co‐cultivation of microalgae and bacteria has been shown to benefit the biosynthesis of lipids, carbohydrates and proteins (Cho et al., 2015, 2019; Perera et al., 2021).

From an industrial point of view, the presence of other microorganisms (e.g., pathogenic fungi, viruses and bacteria) is often associated with a loss of algal biomass and decreased purity of co‐products (Lam et al., 2018; Molina et al., 2019). Therefore, the mechanisms by which these species interact need to be thoroughly understood before a co‐culture strategy between algae and bacteria can be implemented on a commercial scale.

Currently, the majority of scientific publications state that these types of interactions or their specific functionality (e.g., nutrient removal, metabolite production or flocculation) between microalgae and bacteria are “species‐specific” (Cho et al., 2015; Eigemann et al., 2013; Ferro et al., 2019; Krug, Morauf, et al., 2020; Lee et al., 2013; Sepehri et al., 2020). The most commonly proposed mechanisms of algal growth promotion using bacteria are related to the synthesis and secretion of vitamins, siderophores, plant hormones and O_2_—CO_2_ exchange by bacteria (Table S1).

In contrast, the influence of environmental factors (e.g., cultivation conditions) and the physiological state of the microorganism (e.g., growth stage) on interspecies interactions remains undefined. Nevertheless, some studies documented that the nature of the interspecies interactions is not permanent and depends on many factors rather than being solely species‐specific (Sapp et al., 2007). Evidence was found that the mutualistic interaction between algae *Emiliania huxleyi* and *Phaeobacter gallaeciensis* can turn into a parasitic one. The mechanism of action is initiated by the release of senescence signalling molecules (e.g., cell wall lignin monomers, p‐coumaric, sinapic and ferulic acids) by the ageing algae, which induce *P. gallaeciensis* to synthesize new algicides, the roseobacticides (Seyedsayamdost, Carr, et al., 2011; Seyedsayamdost, Case, et al., 2011). Another factor is temperature‐ and salinity‐induced changes in the bacterial and archaeal communities and their (mutualistic or competitive) activities during marine *Dinoflagellate* bloom progression (Zhou et al., 2018). Furthermore, temperature‐induced pathogenicity was observed between *Ruegeria* sp. and *Emiliania huxleyi* (Mayers et al., 2016). Shifts in interactions have also been observed due to changes in the chemical composition of the environment. Supplementation of the medium with tannic acid (TA) increased bacterial abundance, which had a negative effect on the growth of the alga *Desmodesmus armatus* and the diatom *Stephanodiscus minutulus* in comparison with conditions without TA. The scientists suggested that the direct effect of the allelochemical polyphenol was the ability of bacteria to use TA as a carbon and energy source (Eigemann et al., 2013). A similar effect (shift from mutualism to parasitism) was observed among *Pseudomonas* sp. and *C. vulgaris* when an additional organic carbon source (glucose) was introduced (Guo & Tong, 2014). The effect of the N source was also observed. The growth efficiency of *C. vulgaris* co‐cultured with *Microbacterium* sp. varied with changes in the ratio of the N source in the medium. This was suggested to be due to the preference of *Microbacterium* sp. for ammonium over NO_3_ or NO_2_ as an N source for growth (Cho et al., 2015). Taken together, the evidence suggests that the effect of species specificity on interactions can be overridden not only by physiological changes in the microorganisms but also by environmental conditions.

In this study, bacteria of the genus *Delftia* sp. were found in a suspension of *Chlorella vulgaris* (UTEX 395) cultured in panel photobioreactors for research at Arizona State University. It has been hypothesized that the source of this contaminant was the incompletely sterilized water used in large‐scale cultivation. However, the fact that the bacteria were strongly attached to *Chlorella* (it was necessary to repeat the purification process multiple times before the bacteria could no longer be detected) and that no reduction in algal biomass was recorded suggests a potentially close interaction between the two species. *Delftia* spp. are known to be versatile environmental microorganisms found in a variety of habitats including fresh and marine water, soil, rhizosphere, plants, clinical samples and activated sludge (Camargo et al., 2014; Chen et al., 2019; Han et al., 2005; Jørgensen et al., 2009; Maisuria & Nerurkar, 2015; Prabaningtyas et al., 2021; Roy & Roy, 2019; Shetty et al., 2015; Ubalde et al., 2012; Wang et al., 2022). Although there are limited studies confirming their presence in algal environments, this does not negate the potential for interactions with algae. Barott et al. (2011) identified *Delftia* sp. as part of the microbial community of benthic reef algae and the reef‐building coral *Montastraea annularis*. Other studies have shown the presence of *Delftia* sp. in activated sludge consortia observed in Chinese and US wastewater treatment plants (Chen et al., 2019; Wang et al., 2022). These activated sludge consortia have been shown to stimulate algal growth and improve wastewater treatment efficiency (COD and nitrogen removal) when co‐cultured with algae such as *Scenedesmus* sp. and *Chlorella* sp. Despite these findings, the specific nature of the interaction between *Delftia* sp. bacteria and algae remains unexplored. Therefore, the main objective of this study was to investigate the relationships between *C. vulgaris* and bacterial contaminants and to have a better understanding of these interactions in general, so that practical applications such as open pond culture could benefit. Since there is no specific medium for assessing interspecies interactions, we studied both BG‐11 and TAP media. BG‐11 was chosen because it is widely used as a standard medium for autotrophic cultivation (where the main carbon source is inorganic), with practical applications in the initiation of lipid synthesis to produce biofuels (Pandey et al., 2023; Vishwakarma et al., 2019; Yadav et al., 2023). Additionally, BG‐11 was the original medium from which the bacteria were isolated in our study. The TAP medium, which is used for heterotrophic/mixotrophic growth (the main carbon source is organic and diurnal lighting), was chosen based on our experience and previous studies, which have shown that it provides a higher multiplication rate and a satisfactory cell density for further isolation of algal high‐value compounds (Castillo et al., 2021; EL‐Moslamy et al., 2016; Ward & Rehmann, 2019). To investigate the potential reason for the differences observed in algal growth under TAP and BG‐11 co‐culture experiments with *Delftia* sp., we employed an additional medium (BG‐11 + acetic acid, also referred to as BG + AA), which could reveal the impact of organic carbon source on algal and bacterial population dynamics. The influence of medium composition, particularly carbon source, on algal–bacterial interactions is a topic of considerable research interest. Typically, supplementation with organic carbon sources has been investigated as a way to achieve improvements in algal growth and productivity, as demonstrated by enhancements in biomass yield and lipid content in *C. vulgaris* (Li et al., 2022; Yeh et al., 2012; Yu et al., 2019). However, the specific effects of acetic acid supplementation in co‐cultures remain relatively unexplored.

## EXPERIMENTAL PROCEDURES

### Isolation and cultivation of microalgae‐associated bacteria

The bacterial isolate used in this study was purified from a *C. vulgaris* (UTEX 395) sample grown in a flat panel photobioreactor at the Arizona Center for Algae Technology and Innovation (AzCATI), at Arizona State University, USA, through serial dilution plating on a nutrient medium. Axenic bacteria were cultured in tryptic soy broth (TSB, peptone from casein 17.0 g l, peptone from soya bean 3.0 g l, D‐(+)‐glucose 2.5 g l, NaCl 5.0 g l and K_2_HPO_4_ 2.5 g l) and in solid TSB Petri dishes (+1.5% [w v] bacteriological grade agar). One colony from a pre‐grown Petri dish was used for the liquid pre‐culture of bacteria, which were aerobically incubated in an orbital shaker at 30°C for 24 h.

### Identification of bacterial isolate

#### Measurement of phenotypic–biochemical parameters

To characterize purified species, phenotypic (Gram staining (Merck KGaA, Germany), cell shape and mobility) and biochemical (RapID™ NF Plus Panel, Remel, USA) analyses were performed (Pitt & Barer, 2012; Staradumskyte & Paulauskas, 2014). The biochemical test panel consists of different tests that are based on the microbial degradation of specific chromogenic substrates (for the full composition of the panel tests, see Table S2).

#### Evaluation of indole‐3‐acetic acid (IAA) synthesis

To assess the ability of bacteria to synthesize IAA, bacteria were grown in a TSB medium with or without 0.2%–1% (w v) L‐tryptophan and cultured under the conditions (see Section 2.1). Subsequently, 1 mL of the suspension was centrifuged at 3000 g for 10 min, and 0.5 mL of the supernatant was mixed with 0.5 mL of Salkowski's reagent (2 mL of 0.5 M iron [III] chloride and 98 mL of 35% perchloric acid) (Glickmann & Dessaux, 1995; Ubalde et al., 2012). After 30 min of incubation in darkness, the development of the colour (pink or red) was quantified using a spectrophotometer at 530 nm and compared with the IAA standard curve.

#### 16S ribosomal gene amplification and sequencing

The 16S ribosomal gene amplification was carried out by colony polymerase chain reaction (PCR) as follows (Woodman et al., 2016): Fresh bacterial colonies were removed from a solid TSB plate with a sterile toothpick and resuspended in 200 μL of sterile deionized water. The PCR tube was boiled for 15 min, at 99°C using FlexCycler Thermal Cycler (Analytik Jena AG, Germany). When the solution was cooled to room temperature, a tube was centrifuged for 1 min at 10,000 g. The supernatant was transferred to a new PCR tube where it was mixed with a PCR mixture. The PCR mixture consisted of 0.02 U μL of Phusion™ High‐Fidelity DNA Polymerase, 1× Phusion™ HF Buffer (5X), 200 μM of dNTP, 0.5 μM of each primer, 3% DMSO, 1 μL of template DNA and 9.9 μL of Milli‐Q water in a final volume of 20 μL. 16S rRNA gene was amplified using primers 27F (5′‐AGA GTT TGA TCM TGGCTC AG‐3′) and 1495R (5′‐CTACGGCTACCTTGTTACGA‐3′) (Kuisienė et al., 2007). The DNA fragment for sequencing was purified from a PCR mixture using GeneJET Gel Extraction and DNA Cleanup Micro Kit (Thermo Fisher Scientific, Lithuania) following the instructions in the user manual. DNA concentration was measured using a NanoDrop UV–Vis spectrophotometer (Thermo Fisher Scientific, USA). Additionally, DNA electrophoresis was performed in 0.9%–1.3% agarose gels prepared in 1 × Tris–acetate–EDTA (TAE) buffer using a horizontal electrophoresis system at a voltage of 7.6 V cm. Agarose gel was stained with ethidium bromide (0.5 μg mL) and visualized with a UV transilluminator (VWR International, UK).

Subsequently, the DNA was sent to the DNA Sequencing Center of the Institute of Biotechnology, Vilnius University, Lithuania, for sequencing. The resulting DNA sequences were then aligned using Geneious Prime software (version 2023.0.2) with the MAFFT v7.490 (Katoh et al., 2002; Katoh & Standley, 2013) multiple alignment tool, which applied the auto‐algorithm with specific parameters including a scoring matrix of 200PAM/*k* = 2, a gap open penalty of 1.53 and an offset value of 0.123. After sequence alignment, the resulting sequences were compared with previously published sequences available in the GenBank NCBI database using the BLAST. This step was crucial to identify similarities and differences with known sequences. To clarify the evolutionary relationships between the sequences, a phylogenetic tree was constructed. This was done using the MrBayes plugin (v3.2.6) (Huelsenbeck & Ronquist, 2001) and the multiple alignment file with a JC69 substitution model, gamma rate variation, gamma categories of 4 and *Variovorax paradoxus* as the outgroup. Markov chain Monte Carlo (MCMC) settings were as follows: chain length = 1,100,000; subsampling freq = 200; heated chains = 4; burn‐in length = 100,000; heated chain temp = 0.2 and random seed = 16,656. Prior settings were as follows: GammaDir (1,0.1,1,1) with exponential = 10 shape parameter.

#### 16S ribosomal gene restriction analysis

After PCR amplification, the amplified 16S ribosomal gene fragment was digested with *Eco*RI and *Ehe*I enzymes (Urata et al., 2004). The digestion reaction contained 10 μL of PCR product, 1 × Buffer Tango (10×), 1 μL of restriction enzyme (*Eco*RI and *Ehe*I) or 1 μL of each and Milli‐Q water to a final volume of 20 μL. The DNA hydrolysis mixture was incubated at 37°C for 2 h. The resulting restriction fragments were analysed by electrophoresis on an agarose gel, as mentioned in Section 2.2.3.

#### Matrix‐assisted laser desorption/ionization–time‐of‐flight mass spectrometry (MALDI‐TOF MS)

Before MALDI‐TOF MS analysis, bacterial isolates were freshly inoculated on a solid TSB plate and cultivated for 24 h at 30°C (Panda et al., 2014). MS analysis was performed on an Autoflex MALDI‐TOF mass spectrometer (Bruker Daltonics, Germany) using flexControl 3.4 software (Bruker Daltonics, Germany) at the National Public Health Surveillance Laboratory (NPHSL).

#### Microalgal culture

*C. vulgaris* (SAG strain 211–12, University of Göttingen) pre‐culture was grown in 1 × Tris–acetate–phosphate (TAP) medium in 400‐mL Erlenmeyer glass flasks as described in detail in a previous study (Krust et al., 2022). The TAP medium consists of 17.49 mM acetic acid; 19.98 mM Tris; 7.01 mM NH_4_Cl; 0.4 mM MgSO_4_ × 7H_2_O; 0.34 mM CaCl_2_ × 2H_2_O; 0.63 mM K_2_HPO_4_; 0.4 mM KH_2_PO_4_; 0.17 mM EDTA; 0.18 mM H_3_BO_3_; 0.08 mM ZnSO_4_ × 7H_2_O; 0.03 mM MnCl_2_ × 4H_2_O; 0.02 mM FeSO_4_ × 7H_2_O; 0.007 mM CoCl_2_ × 6H_2_O; 0.006 mM CuSO_4_ × 5H_2_O and 0.001 mM (NH_4_)_6_Mo_7_O_24_ × 4H_2_O pH 7.0 (Hutner et al., 1950). After inoculation to an OD of 0.1, measured at 750 nm, the axenic algal suspension was cultured at 21 ± 1°C under illumination with fluorescent lamps at a photosynthetic photon flux density of 90 μmol m^−2^ s^−1^, 16:8‐h light:dark cycle and shaking at 150 rpm (standard analogue shaker, VWR International, Leuven, Belgium) until harvested at the stationary growth phase, 7 d.

#### Evaluation of the effect of bacteria on microalgae by means of co‐cultivation experiments

To examine the effects of bacteria on *C. vulgaris*, microalgal growth was compared in the presence and absence of bacteria under different growth conditions, TAP, BG‐11 and BG‐11+ acetic acid. The ingredients of the TAP medium are listed in Section 2.3, whereas the main nitrogen source is ammonium in NH_4_Cl from 7.01 mM TAP salts. The BG‐11 medium is composed of 17.65 mM NaNO_3_; 0.23 mM K_2_HPO_4_; 0.3 mM MgSO_4_ × 7H_2_O; 0.25 mM CaCl_2_ × 2H_2_O; 0.19 mM Na_2_CO_3_; 0.03 mM citric acid; 0.02 mM ammonium ferric citrate; 0.002 mM Na_2_‐EDTA; 0.05 mM H_3_BO_3_; 0.009 mM MnCl_2_ × 4H_2_O; 0.0008 mM ZnSO_4_ × 7H_2_O; 0.002 mM Na_2_ MoO_4_ × 2H_2_O; 0.003 mM CuSO_4_ × 5H_2_O and 0.0002 mM Co(NO_3_)_2_ × 6H_2_O (Cho et al., 2013), with 17.65 mM nitrate from NaNO_3_ and 0.02 mM ammonium from ammonium ferric citrate as the nitrogen source and 0.02 mM citrate from ammonium ferric citrate as the organic carbon source. BG + AA is a BG‐11 medium containing 17.49 mM acetic acid, as in TAP medium, to compensate for the lack of organic carbon. Each flask (TAP, BG‐11 and BG + AA) was inoculated with axenic *C. vulgaris* (initial optical density 0.1, at 750 nm) and bacterial culture (initial optical density 0.01, at 600 nm). All flasks were incubated under the conditions (see Section 2.3) without CO_2_ supply for 7 d. The change in optical density was measured daily using a spectrophotometer (Halo RB‐10, Dynamica Scientific Ltd., UK) at a wavelength of 750 nm for microalgae and 600 nm for bacteria. Algal and bacterial cell concentration, algal viability and algal mortality were determined using separate staining methods of SYTO® 9 (cell‐permeable nucleic acid dye, Invitrogen by Thermo Fisher Scientific) and YO‐PRO‐1®‐1 (cell‐impermeable nucleic acid dye, Invitrogen by Thermo Fisher Scientific). Algal cell metabolic activity was monitored by measuring the intensity of the fluorescein signal, which is a product of hydrolysed fluorescein diacetate (FDA; non‐fluorescent, cell‐permeable esterase substrate, Invitrogen by Thermo Fisher Scientific). Briefly, the freshly collected samples were mixed with SYTO 9, FDA or YO‐PRO‐1 (final dye concentration 0.5 μM) and incubated in the dark for 5 min (10 min for YO‐PRO) (Krust et al., 2022). After incubation, stained samples (with SYTO 9, YO‐PRO‐1 or FDA) were diluted by a factor of 10 in sterile TAP medium. The fluorescence signals of dyes (a green fluorescence bandpass filter of 530 ± 30 nm [BL1]) and chlorophyll‐*a* autofluorescence (a far‐red bandpass filter of 695 ± 40 nm [BL3]) were quantified using a flow cytometer (FCM, Attune™ Nxt, Thermo Fisher Scientific, excitation at 488 nm). The volume of the sample ranged between 50,000 and 100,000 events with a flow rate of 25 μL min. After measurement, the data were displayed as dot plots showing BL3‐H versus BL1‐H (in logarithmic mode). With the help of chlorophyll‐*a* autofluorescence, the different populations were gated (algal cells were distinguished from bacteria) and a number of events were recorded. Cells with a signal greater than 10,000 (BL3‐H) were defined as microalgae (hereafter referred to as chlorophyll positive, Chl+), and cells with a signal less than 10,000 were defined as non‐algae (hereafter referred to as chlorophyll negative, Chl−). Cells with a signal greater than 10,000 (BL1‐H) were defined as positive for a specific dye (hereafter referred to as SYTO 9+/YO‐PRO‐1+/FDA+), and the rest were defined as negative for a specific dye (hereafter referred to as SYTO 9−/YO‐PRO‐1−/FDA−). The data obtained were expressed in several ways: Algal mortality (%) is determined by calculating the proportion of dead algal cells, labelled with Chl+YO‐PRO‐1+, against the total number of algal cells stained with Chl+SYTO9+. Similarly, for bacteria, Chl− is used to express bacterial mortality. Viable algal cell concentration is determined by subtracting the algal mortality (%) from the total number of algal cells stained with Chl+SYTO9+ (with Chl−, respectively, for expression of viable bacterial cell concentration). Algal viability (%) is the ratio of Chl+FDA+‐stained cells to the total number of Chl+SYTO9+‐stained algal cells. To validate the flow cytometer data, additional bacterial growth detection tests including colony‐forming units, cell count using CASY Cell Counter & Analyzer (OMNI Life Science, Germany) and optical density measurements were performed.

#### Nutrient determination

Changes in medium composition were determined daily from the start of inoculation until the last day of the experiment. Briefly, freshly collected samples were centrifuged and the supernatant was immediately analysed for NH_3_ (Merck KGaA, Germany), ${\mathrm{NO}}_{3}^{–}$ (Merck KGaA, Germany), ${\mathrm{NO}}_{2}^{–}$ (Merck KGaA, Germany) and acetic acid (Megazyme, Ireland) according to the manufacturer's protocol. In addition, the IAA content was measured as described in Section 2.2.2.

#### Microscopic imaging

To observe bacterial attachment to algae or their ability to form agglomerate, samples were microscopically analysed by bright‐field and fluorescence microscopy (Imager.M2 Axio, LD Plan‐Neofluar 40x, Zeiss, Göttingen GER). Images were captured with a 5‐megapixel microscope camera (Axiocam 105 colour, Zeiss Göttingen, Germany) using ZEN Pro acquisition software. To avoid deformation and crushing of the cell aggregates, the samples on the slides were not covered with a coverslip when imaging the cell aggregates. In this case, the images were taken within a few minutes (3–5 min) to avoid changes due to drying of the samples. To assess the percentage of aggregated cells, a cell counting chamber (Neubauer, Marienfeld, Germany) with a depth of 100 μm was used. Samples were filtered through a 100‐μm nylon membrane prior to filling the chamber to exclude aggregates larger than 100 μm in diameter. After taking the microscopic images, the projection area on the image plane (*A*_*P*_) and the minor axis of the aggregates (2·*r*) were measured using the AxioVision software measuring tools (Zeiss, Göttingen, Germany). The number of cells in the aggregate was determined according to Martin et al. (1997). Briefly, the estimated volume of the aggregate is calculated according to the following formula: *V*_aggregate_ = 4/3·*A*_*P*_·*r*, where *r* is half of the minor axis. In this model, the aggregates are modelled as an assembly of touching spheres, representing single cells with a known average volume. Assuming a packing density of 0.632, which is the average between the face‐centred cubic lattice packing (spheres occupy 0.7405 of the total volume) and the 3D simple cubic lattice packing (spheres occupy 0.5235 of the total volume), the number of cells in each aggregate can be calculated using the following formula: *N*_in aggregate_ = 0.632·*R*, where *R* is the ratio between the volume of the aggregate and the average volume of the single cells. After estimating the number of cells in each aggregate and the number of single cells in the recorded fields (corresponding to a total volume of 1.8 μL sample), the fraction of cells clustered in aggregates and thus the percentage of cell aggregation (% aggregation) can be determined. The % aggregation was determined daily while growing *C. vulgaris* co‐cultured with bacteria in different media.

### Statistical analysis

All experiments were performed at least in triplicate. Mean values and standard deviations were calculated. The results were evaluated by one‐way analysis of variance (ANOVA) using Excel software with a statistical significance of *p* <0.05.

## RESULTS

### Bacterial identification

#### Morphologic and phylogenetic characteristics of the species

First, a phenotypic analysis was performed to determine the origin of the bacteria purified from *C. vulgaris* cultures (UTEX 395) used in the flat‐plate photobioreactor studies at Arizona State University. The purified colonies on the plate were white, smooth and slightly convex with irregularly scattered edges. Cells observed microscopically were motile, short, rod‐shaped and Gram‐negative.

**FIGURE 1:**
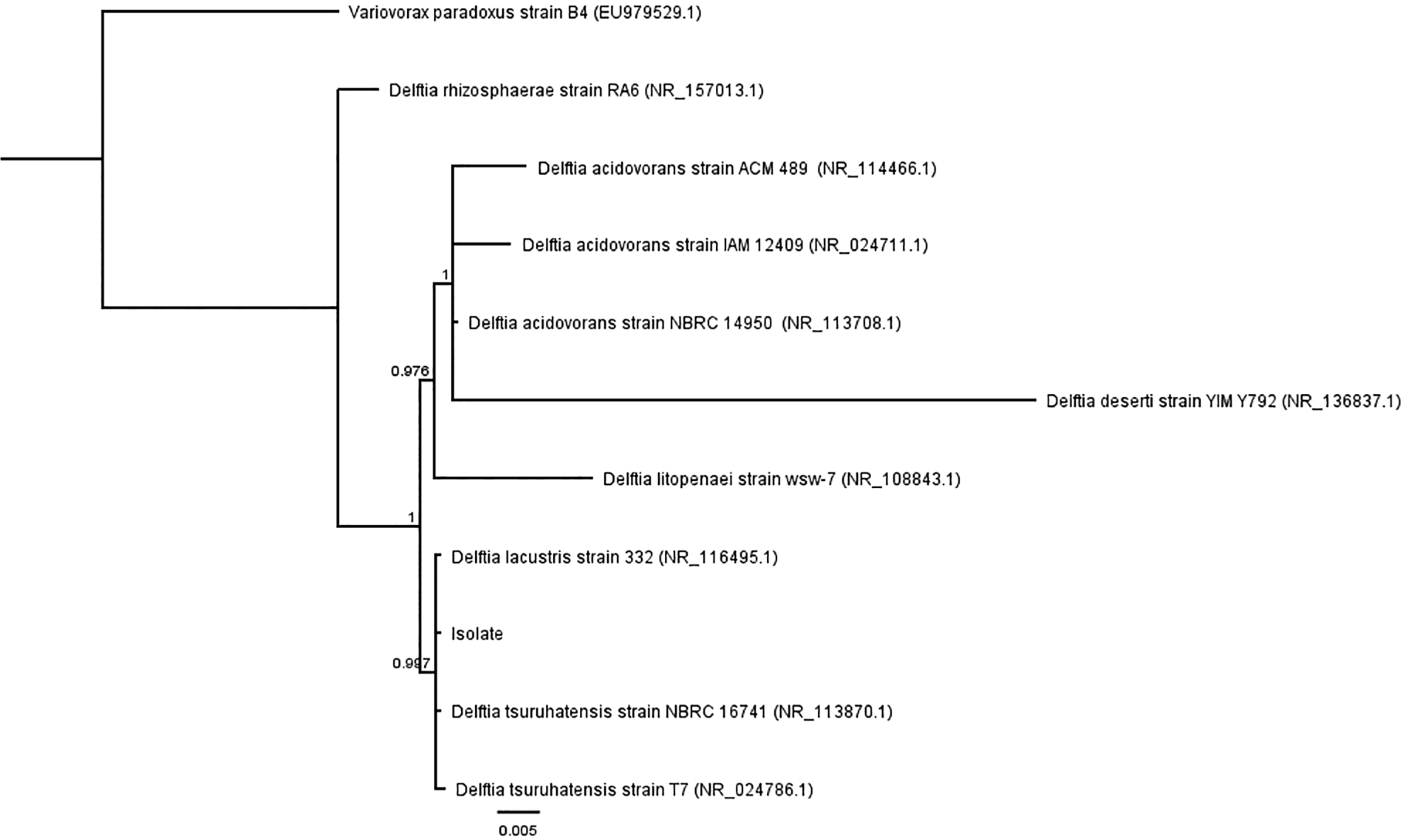
Bayesian tree of *Delftia* species based on 16S rDNA sequences. The tree was rooted with *Variovorax paradoxus* as an outgroup. GenBank accession numbers of the operational taxonomic units are shown in parentheses. Posterior probability is indicated on the branches. Scale bar indicates substitutions per site.

Phylogenetic analyses based on 16S rRNA gene sequences revealed that an unknown strain was closely related to *Delftia lacustris*, *Delftia tsuruhatensis* and *Delftia acidovorans* (98% sequence similarity). Therefore, type species of *Delftia* genus were selected and a phylogenetic tree was constructed (Figure 1). Analysed bacterial strain formed a deep phyletic cluster with *D. tsuruhatensis* (GenBank accession nos. NR 113870.1 and NR 024786.1) and *D. lacustris* (GenBank accession no. NR 116495.1).

**FIGURE 2:**
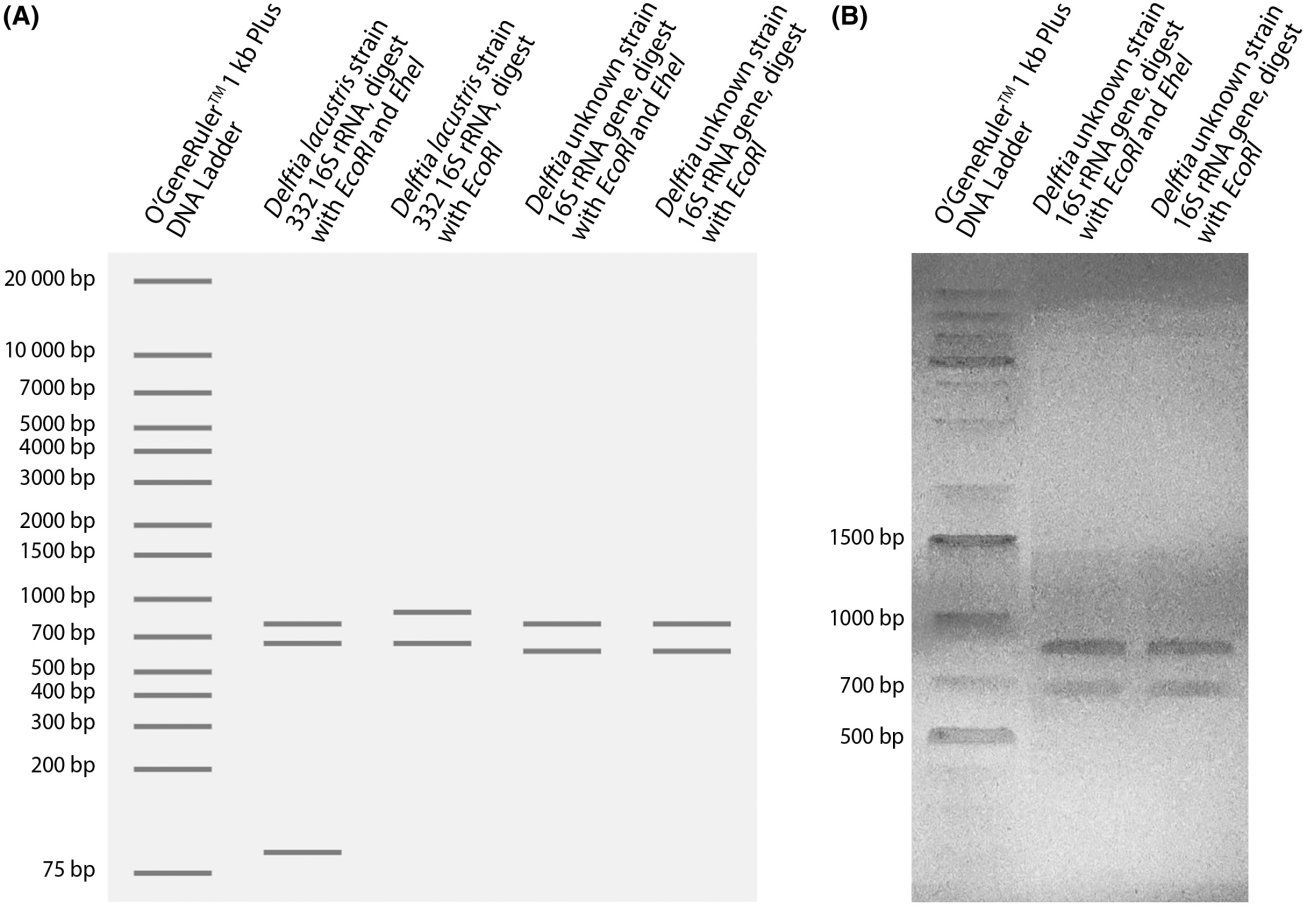
Virtual gel electrophoretic profiles of *D. lacustris* 332 and isolated strain 16S rRNA gene sequences after digestion with *Eco*RI and *Ehe*I enzymes (A). Experimental gel electrophoretic profiles of the isolated strain 16S rRNA gene after digestion with *Eco*RI and *Ehe*I restriction endonucleases (B).

Furthermore, bioinformatics data analysis revealed that the main difference between strains (*D. lacustris* 332 and *D. tsuruhatensis* NBRC 16741) was at the 1446–1450 alignment position, where only *D. lacustris* has an *Ehe*I site. Therefore, to verify whether the isolated bacteria belong to *D. lacustris* 332, the PCR product was digested with *Eco*RI and *Ehe*I restriction enzymes. The data denied the given theory (Figure 2), suggesting that the isolated bacteria are phylogenetically more closely related to *D. tsuruhatensis*.

Since the phylogenetic analysis of the isolate showed two potential candidates within the genus *Delftia*, enzymatic digestion eliminated *D. lacustris* as a candidate. In order to validate the results further, a MALDI‐TOF MS analysis was carried out. The results obtained, logarithmic values (scores) of *D. acidovorans* (2.08) and *D. lacustris* (2.06), were greater than 2.00, indicating ‘high confidence identification’ as a *Delftia* species.

#### Biochemical characteristics

As the results of the MALDI‐TOF MS and 16S rRNA gene sequence partially overlapped, biochemical analysis was performed (Table S2). The data showed that the isolate shared many characteristics common with its closest relatives *D. tsuruhatensis* and *D. acidovorans*. All were positive for lipid hydrolysis and nitrate utilization and negative for arginine, N‐acetyl‐β‐D‐glucosaminide, α‐ and β‐glucoside and β‐D‐galactosidase hydrolysis, D‐glucose utilization and indole formation. The main differences between the strains mentioned above were phosphoester, proline and pyrrolidine hydrolysis.

#### Synthesis of IAA by bacteria

To assess the synthesis of IAA by *Delftia* sp., we used a TSB medium supplemented with L‐tryptophan, as a precursor to the IAA. After 24 h of cultivation, chemical reaction of the supernatant with Salkowski's reagent resulted in colour development (pink colour appeared). It was observed that with increasing concentration of L‐tryptophan (2%–10% [w v]) in the medium, the intensity of the colour absorption at 530 nm increased by 120%. According to the performed standard IAA curve (*R* ^2^ = 0.9666), it was estimated that the bacteria were capable of synthesizing 12–26 mM IAA during 24 h cultivation, leading to the conclusion that *Delftia* sp. can synthesize the phytohormone IAA.

IAAs were also evaluated in co‐culture experiments with algae and bacteria. Daily measurements of samples across different culture conditions showed the absence of IAA. Two potential factors could explain the absence of phytohormone in algal–bacterial co‐culture samples. First, IAA synthesized by the bacteria may have been produced at concentrations below the detection limit of the employed measurement method. Second, if IAA was indeed synthesized, it may have been rapidly absorbed by the algae, further complicating its detection in the samples.

#### Impact of bacteria on C. vulgaris growth

##### Results of microscopic observations

**FIGURE 3:**
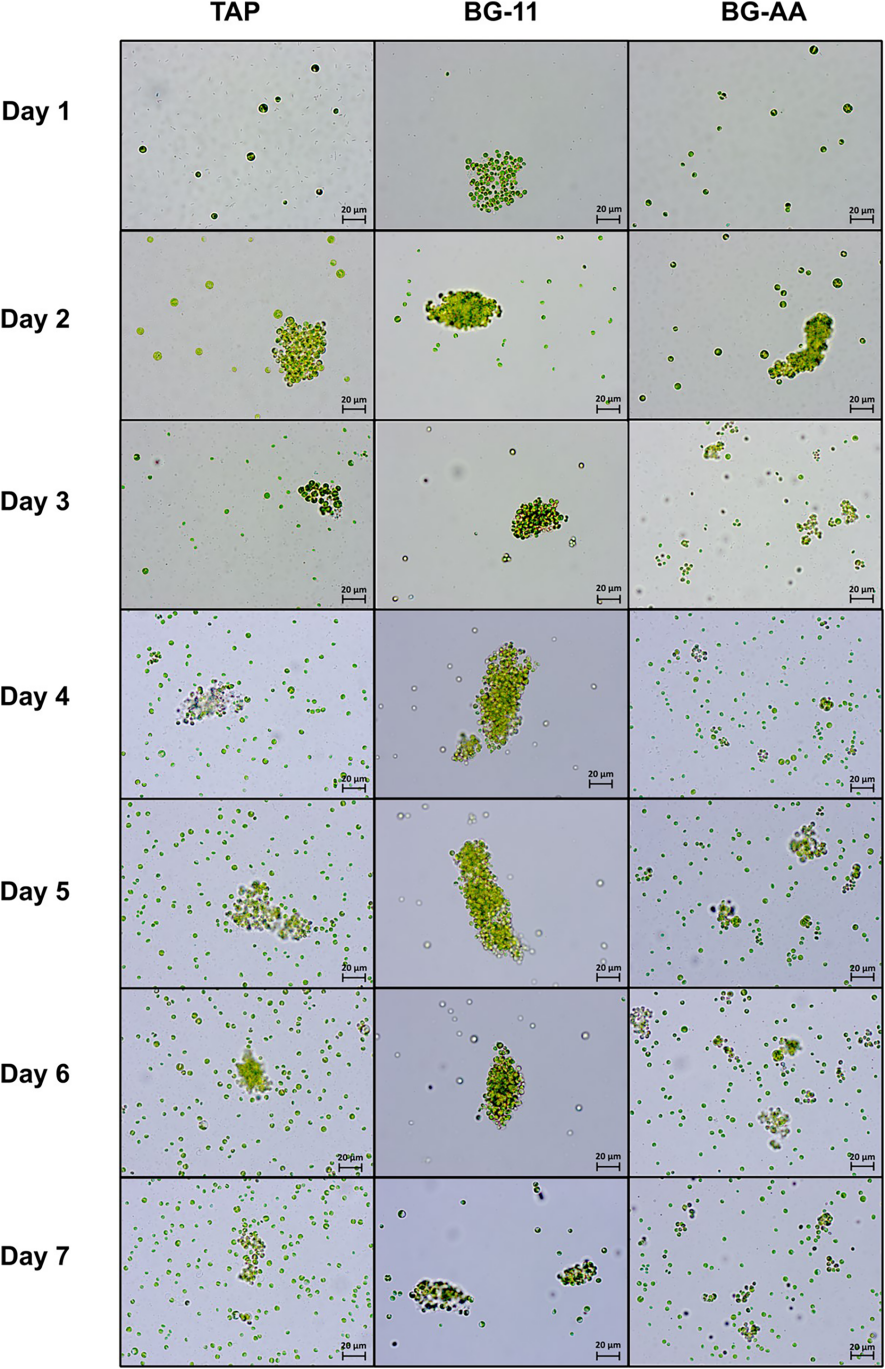
Light microscopy images of co‐culture samples cultivated under TAP (first column from the left), BG‐11 (second column) and BG + AA (third column) conditions for 7 d.

In co‐cultures of algae and bacteria, the first notable observation was the presence of algal agglomerates, which were most noticeable at the beginning of cultivation, from the 1st d to the 4th d. In contrast, a lower algal aggregation was observed in algal monoculture sample. To assess the relationship between the presence of bacteria and the formation of agglomerates, the co‐culture samples were analysed daily using a light microscope. The results showed that the type and size of agglomerates varied depending on the medium. This is illustrated in Figure 3 using some representative selected images. In the TAP and BG + AA co‐culture, the determined percentage of aggregated algae increased during the first days from 25%–40% (4 h after inoculation) to 50%–60% (after 2–3 d; see Figure S1). However, from 4th d, the aggregates became smaller and were over‐dominated by newly proliferated algal cells. Especially in the BG + AA medium, the remaining clusters consisted mainly of parent cells (*C. vulgaris*) trapped in the division phase. During this phase, the cell wall of the parent cell ruptures, releasing the daughter cells. However, it appears that the release of some younger cells was interrupted, causing their cell walls to become entangled with those of other cells in the same division phase. In BG‐11 medium, the aggregates showed the densest cell packing and the highest % of aggregation (over 70%), which remained unchanged over the 7 days of cultivation. In addition, the TAP and BG + AA co‐culture samples were composed of a higher proportion of viable, motile bacteria (colourless shadows, smaller than the algae) than in the BG‐11, where the presence of bacteria was significantly lower.

##### Investigation of the growth of algae and bacteria in co‐cultivates under various conditions

To investigate the effect of our *Delftia* isolate (hereafter described as *Delftia* sp.) on microalgal growth under different cultivation conditions, *C. vulgaris*, *Delftia* sp. and mixed suspension of algae and bacteria were cultured in TAP, BG‐11 and BG + AA conditions. By staining samples separately with different fluorescent dyes (SYTO 9, YO‐PRO‐1 and FDA) and using an FCM, subsets of bacteria and microalgae were differentiated and the number of viable cell concentrations, algal viability and algal or bacterial mortality were determined. Additionally, optical density, colony‐forming units, cell count using the CASY Cell Counter and pH measurements were used to validate FCM results.

**FIGURE 4:**
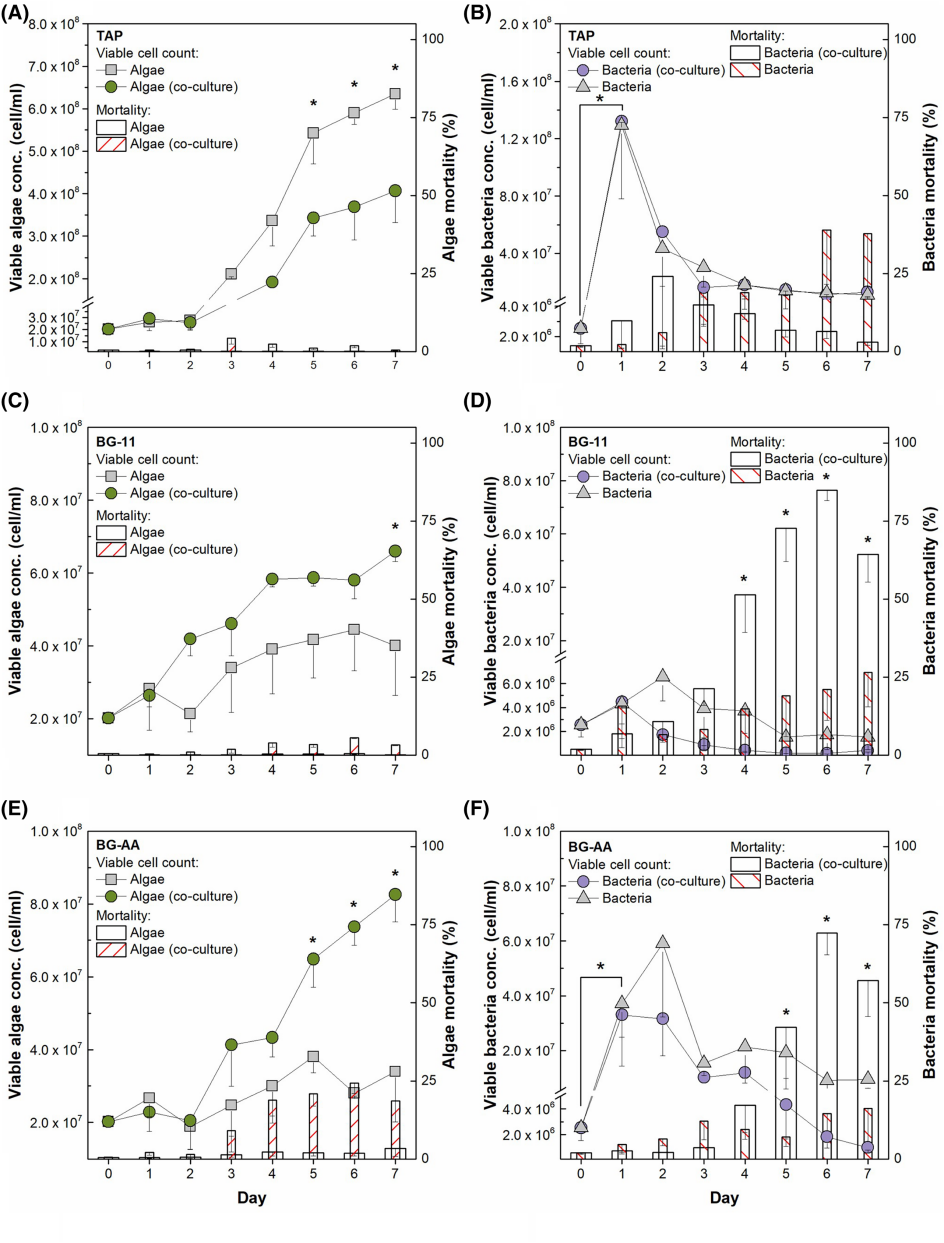
Co‐culture of *C. vulgaris* and *Delftia* sp. in TAP (A, B), BG‐11 (C, D) and BG + AA (E, F) media. In graphs A, C and E, the left Y‐axis indicates viable algal cell concentration, and the right Y‐axis indicates algal mortality, as described in 2.4. In graphs D, E and F, the left Y‐axis indicates viable bacterial cell concentration, and the right Y‐axis indicates bacterial mortality. Initial (at day 0) algal cell concentration is around 2 × 10^7^, and initial bacterial concentration is 2.5 × 10^6^. Significant differences (where *p* <0.05) between data points or columns within a single day are indicated by *, and significant differences between two different days are indicated by brackets with *.

The results indicated that the presence of *Delftia* sp. bacteria has a negative effect on *C. vulgaris* growth in the TAP medium (Figure 4A). The evaluation of the viable cell concentration results showed that *Chlorella* in the co‐cultivation (hereinafter Algae [co‐culture]) starts to fall behind from the moment when the growth of the control sample (hereinafter Algae) shifts from the lag phase to the exponential phase. It was also observed that the co‐cultured algal sample reached the stationary phase at the same time (5d) as the control sample. Furthermore, the results of algal viability (metabolic activity) and mortality showed that there was no significant difference between *Chlorella* populations grown with and without bacteria (Figure 4A, Figure S3B). There was only a reduced number of microalgal cells compared to the control sample. Optical density measurements also indicated that algal cell numbers decreased under the co‐culture conditions (Figure S3A).

However, regardless of whether co‐cultured with algae or cultured alone (hereinafter Bacteria and Bacteria [co‐culture]), bacterial concentrations were notably higher when cultivated in the TAP medium (Figure 4B). The ability of the bacteria to multiply under TAP conditions was additionally confirmed by various measurements of *Delftia* sp. growth during the first 24 h of cultivation when the TAP medium was inoculated with bacteria alone (Figure S2).

In the BG‐11 medium, the presence of *Delftia* sp. bacteria had a positive effect on *C. vulgaris* growth (Figure 4C). An increase in cell concentration (hereafter Algae [co‐culture]) was observed from day 2. However, the concentration of algae in the BG‐11 medium was about 10 orders of magnitude lower than in the TAP medium. In addition, no significant bacterial growth was observed, whether co‐cultured with algae or cultured alone, under BG‐11 conditions (Figure 4D, Figure S2). Only co‐cultured bacteria were significantly more likely to die than those grown alone (Figure 4D).

A completely unexpected effect of *Delftia* sp. on the growth of *C. vulgaris* was observed when BG‐11 was supplemented with acetic acid (Figure 4E). The presence of bacteria resulted in a doubling of the algae, while the cell concentration in the control sample remained similar to that of the BG‐11 medium. However, supplementation with acetic acid resulted in bacterial growth as observed under TAP conditions (Figure 4B,F).

By comparing the co‐culture results, another important finding was observed. Under the BG‐11 and BG + AA conditions, the co‐cultured algal samples became more alkaline (pH >9.5) than the samples under the TAP conditions (Figures S3C, S4C, S5C). Furthermore, under BG + AA conditions, *C. vulgaris* samples (with and without *Delftia* sp.) exhibited the highest increase in pH (more than 10) compared to other cultivation conditions (Figure S5C).

**FIGURE 5:**
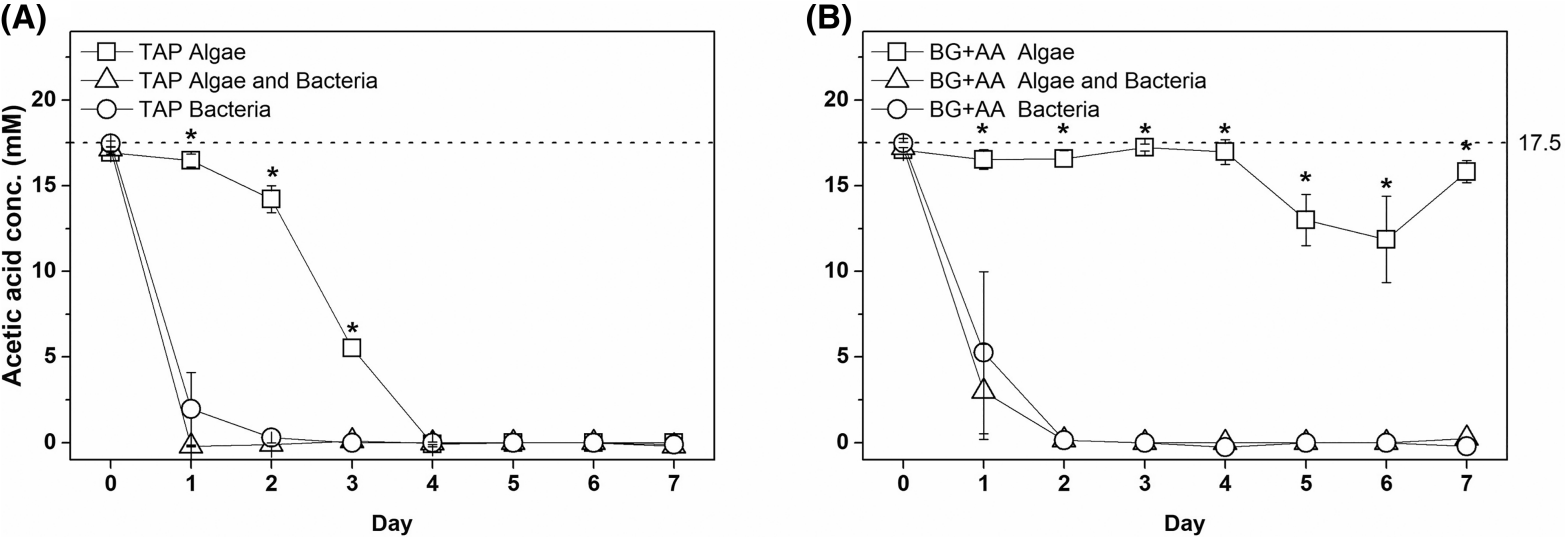
Changes in acetic acid concentration in *C. vulgaris* and *Delftia* sp. grown together under TAP (A) and BG + AA (B) conditions. The dotted horizontal line indicates the initial concentration of the organic carbon compound in the medium, calculated from the composition of the medium. Statistical significance between data points for individual days is indicated by an *when *p* <0.05.

To investigate possible factors that may have contributed to the different growth behaviour of *C. vulgaris* in co‐culture with bacteria, samples were further analysed for changes in medium chemical composition. As intensive bacterial growth and agglomerate formation were only observed under conditions where acetic acid was present, this compound was analysed first. Analysis of the changes in acetic acid concentration during cultivation confirmed that *Delftia* sp. was not only able to assimilate the organic carbon compound but also assimilated it faster than the algae alone (Figure 5). Moreover, *C. vulgaris* did not assimilate acetic acid even when grown on BG + AA medium alone (Figure 5B).

In addition to the uptake of acetic acid, changes in the N compounds (${\mathrm{NH}}_{4}^{+}$, ${\mathrm{NO}}_{2}^{-}$ and ${\mathrm{NO}}_{3}^{-}$) were also observed under the BG and BG + AA conditions (Figures S6 and S7). Under BG‐11 conditions, samples containing *C. vulgaris* started to release increasing amounts of ${\mathrm{NO}}_{2}^{-}$ into the medium over the cultivation period. However, this tendency was not observed in the samples where only *Delftia* sp. was present. The opposite was observed under BG + AA conditions, where all samples showed an increase in ${\mathrm{NO}}_{2}^{-}$, albeit to a lesser extent. The results for ${\mathrm{NH}}_{4}^{+}$ changes were less accurate. Under BG + AA conditions, a significant increase in ${\mathrm{NH}}_{4}^{+}$ concentration was observed during cultivation in the samples containing only bacteria.

## DISCUSSION

An important finding from the co‐cultivation experiments in various growth media is the contrasting impact of *Delftia* sp. on the growth of *C. vulgaris*. In TAP medium, there was a notable negative effect of *Delftia* sp. on the growth of *C. vulgaris*, whereas in BG‐11 and BG + AA, there was better growth of the microalgae, although to a lesser extent than in TAP (Figure 4). This negative interaction is likely driven by nutrient competition, as evidenced by the robust bacterial growth and acetic acid assimilation in TAP and BG + AA media (Figure 4B,F, Figure 5). It should also be noted that citrate, a component of BG‐11 and BG + AA, can also support the growth of *Delftia* sp. as an organic carbon source (Chen et al., 2023; Zhang et al., 2010). However, the low concentration of citrate (0.02 mM compared to 17.49 mM of acetic acid) is likely to be consumed very quickly, as shown by the limitation of exponential bacterial growth in BG‐11 (without acetic acid) to 8 h (Figure S2, BG‐11), and therefore has only a minimal effect on bacterial concentration in BG + AA with acetic acid as the main carbon source. The depletion of acetic acid as an organic carbon nutrient is further supported by the decrease in viable bacterial concentration from 2nd d. Analysis of co‐cultivation dynamics showed that bacterial proliferation was predominantly in the early stages of culture (1st to 2nd d) in all media, with a statistically significant increase in viable cell concentration under TAP and BG + AA conditions (Figure 4B,D,F). From 3rd d, bacterial mortality began to increase and exceeded 50% in the later stages of cultivation in BG‐11 and BG + AA, indicating an increased level of stress, probably due to nutrient limitation. Conversely, in the TAP medium, bacterial mortality peaks only on 2nd and 3rd d, while algal growth is inhibited by bacterial proliferation throughout the cultivation period.

The suggestion that bacteria are under nutrient stress in TAP and BG + AA is also supported by microscopic observations of increased microalgal aggregation during the early stages of co‐culture (Figure 3, Figure S1). In this phase, acetic acid is consumed by bacteria within 1 day (Figure 5). However, cell aggregation diminished as algal cell reproduction accelerated, after 3 d. Under BG‐11 conditions, algal aggregation is induced from the beginning and remains constant throughout the cultivation process, indicating sustained bacterial nutrient stress due to the lack of a major carbon source (only 0.02 mM citrate is available). The literature suggests that the mechanism underlying bacterial‐induced algal cell aggregation could involve direct attachment to neighbouring microalgal cells, although this was not directly observed under the microscope. Alternatively, extracellular proteins or polymer particles released by bacteria under stress conditions, such as nutrient deprivation, could also contribute to the promotion of algal aggregation (Powell & Hill, 2013; Wang et al., 2012). Literature suggests that the genus *Delftia* sp. is able to form agglomerates when exposed to harsh environmental conditions (Afonso et al., 2023; de Gannes & Hickey, 2017). However, verifying the observed differences in *C. vulgaris* aggregation patterns under co‐culture with *Delftia* sp. bacteria and different cultivation conditions requires further extensive research.

Another significant finding is that the presence of *Delftia* sp. in the BG‐11 medium positively influenced the growth of *C. vulgaris*. However, the bacteria reached the stationary phase very quickly, around 8 h after inoculation (Figure S2), with a subsequent gradual decrease in the number of viable bacteria (Figure 4D) after 2nd d. It is important to note that the concentration of algae at the end of the cultivation, with or without bacteria, was several times lower in the BG‐11 medium than in the TAP medium, as indicated by OD measurements (Figures S3–S5). This reduction in *C. vulgaris* growth between BG‐11 and TAP may be due to the difference in media composition. First, from a carbon supply point of view, the BG‐11 medium contains low organic carbon (0.02 mM) and promotes autotrophic algal growth. Typically, this type of cultivation results in a much lower algal biomass yield than under heterotrophic or mixotrophic condition (EL‐Moslamy et al., 2016). Second, different forms of nitrogen also affect algal growth. Nitrate, which dominates in the BG‐11 medium, has a higher energy requirement for uptake and assimilation in algal cells compared to ammonium in the TAP medium. In addition, ammonium is a more efficient nitrogen source for microalgal growth (Kumar & Bera, 2020). The lack of nutrients, especially of a sufficient organic carbon source for bacteria in the BG‐11 medium, leads to a low bacterial density during cultivation and even to a significant increase in mortality (Figure 4D) towards the end of cultivation, which can be attributed to starvation stress.

The presence of *Delftia* sp. had a significantly greater positive effect on the growth of *C. vulgaris* under BG + AA conditions compared to BG‐11 conditions (Figure 4E). In co‐cultures, the algal concentration increased twofold compared to the algae grown alone. Observations of acetic acid consumption and population dynamics indicated that algae alone were unable to utilize acetic acid in BG + AA, resulting in the algal concentration yield obtained under BG‐11 conditions (Figure 5). Conversely, acetic acid consumption only occurred in samples containing bacteria, resulting in a statistically significant increase in bacterial concentration (Figure 4F). These results show that algal growth was not increased by the addition of organic carbon sources (such as acetic acid). Instead, acetic acid only contributed to an increase in bacterial concentrations under BG + AA conditions. Therefore, the possible factors leading to an increase in algal growth can only be related to the increased bacterial concentration in the co‐culture. One of the plausible factors leading to enhanced algal growth could be bacterial degradation products that serve as additional nutrients for the algae. One such potential bacterial degradation product is IAA, the synthesis of which has been confirmed for isolated bacteria (3.1.3). The positive effects of IAA on *C. vulgaris* have already been demonstrated in extensive research. These include enhanced growth through interaction with IAA‐producing bacteria such as *Azospirillum brasilense*, increased photosynthetic pigment concentrations, stimulated metabolite accumulation, improved antioxidant activities and enhanced nutrient uptake, particularly of phosphorus (Fathy et al., 2023; Meza, de‐Bashan, & Bashan, 2015; Meza, de‐Bashan, Hernandez & Bashan, 2015; Piotrowska‐Niczyporuk & Bajguz, 2014). However, attempts to detect IAA in co‐cultures have been unsuccessful, although several *Delftia* species are known to synthesize this phytohormone, which promotes the growth and biochemical composition of a wide range of plants, including algae (Roy & Roy, 2019; Ubalde et al., 2012). It is possible that IAA was not detected because the concentrations were below the detection threshold of the method used or that the uptake by the algae was so rapid that detection was unlikely. A second plausible factor that could contribute to accelerated algal growth is the release of ammonium (${\mathrm{NH}}_{4}^{+}$) by the bacteria (Figure S7). The synthesis of ammonium, evidenced by increased ${\mathrm{NH}}_{4}^{+}$ concentrations in samples containing only bacteria under BG + AA conditions, could be related to *Delftia's* ability to fix atmospheric nitrogen or to reduce ${\mathrm{NO}}_{3}^{-}$ and ${\mathrm{NO}}_{2}^{-}$ to ammonium (Chen et al., 2023; Han et al., 2005; Jørgensen et al., 2009; Li et al., 2020; Roy & Roy, 2019). Ammonium is an essential nitrogen source for algae, facilitating protein synthesis and other metabolic pathways (Kumar & Bera, 2020). In contrast, alternative sources of nitrogen, such as nitrate, which is abundant in the BG‐11 medium, require more energy for uptake, and assimilation by the algae does not begin until ${\mathrm{NH}}_{4}^{+}$ is completely depleted (Kumar & Bera, 2020; Naidoo et al., 2019). Therefore, the synthesis of ammonium by *Delftia* sp. bacteria as a more favourable nitrogen source is expected to promote the growth of *C. vulgaris* in co‐culture with *Delftia* sp.

The observations on acetic acid uptake are partially supported by the pH measurements. In cases where acetic acid uptake occurred, there was an increase in pH, reaching 8–8.5 under TAP conditions and 9–11 under BG + AA conditions (Figures S3 and S5). However, it is important to note that algae alone under BG + AA conditions showed an increase in pH even in the absence of acetic acid assimilation. This is partly due to the lack of buffering capacity in BG‐11 (due to the absence of Tris or TES in the BG‐11 medium) and partly due to the predominant use of carbon dioxide by the microalgae, leading to an accumulation of free hydroxide ions (OH^−^) in the medium. In the presence of bacteria, the production of ammonium by the bacteria leads to a further increase in the pH value. This alkalization of the BG + AA medium may have prevented *C. vulgaris* from assimilating acetic acid (Naidoo et al., 2019; Yeh et al., 2012), despite its known ability to use various sugars and weak acids as carbon sources (Cheng et al., 2022; Lacroux et al., 2021; Syrett et al., 1964).

While organic carbon supplementation has shown promise in increasing algal growth and productivity (Li et al., 2022; Yeh et al., 2012; Yu et al., 2019), the specific effects of acetic acid in co‐cultures are not clear. For instance, co‐culture of *Chlamydomonas reinhardtii* algae with *Pseudomonas* sp. bacteria in TAP medium (originally containing acetic acid) resulted in decreased algal growth yet surprisingly increased algal H_2_ production (Fakhimi et al., 2019). However, this effect was not due to the rapid consumption of the carbon source by the bacteria. Instead, the bacteria metabolized acetic acid much more slowly than the algae. Nevertheless, their presence increased O_2_ consumption, delayed acetic acid uptake by *Chlamydomonas* and induced hypoxic conditions that stimulated H_2_ production (Fakhimi et al., 2019). Different effects of acetic acid supplementation were observed between *C. sorokiniana* and the yeast *Saccharomyces cerevisiae*. Algal and yeast co‐cultures under TAP + mannose conditions showed increased algal growth. Here, the positive effect was achieved through mutual carbon and nitrogen exchange, with the yeast fermenting mannose to produce carbon dioxide for use by the microalgae and the microalgae providing nitrogen to the yeast by metabolizing nitrite to ammonium (Naidoo et al., 2019). Numerous additional studies have explored the impact of other dissolved organic carbon sources (Grossart & Simon, 2007; Rier & Stevenson, 2002; Zhang et al., 2022). For example, co‐cultivation of *C. vulgaris* and *Pseudomonas* sp. under photoautotrophic conditions had a positive effect on algal growth. In contrast, glucose supplementation under photoheterotrophic conditions had a negative effect on algae (Guo & Tong, 2014).

In addition to the above‐mentioned properties of *Delftia* sp. that affect the growth of *C. vulgaris*, several studies have highlighted additional mechanisms by which *Delftia* sp. can affect the growth of plants and algae. *Delftia* sp. has been found to be part of the microbial community structure of benthic reef algae and reef‐building coral *Montastraea annularis* (Barott et al., 2011). They have also been found in activated sludge consortia with growth‐promoting effects on algae such as *Scenedesmus* sp. and *Chlorella* sp. (Chen et al., 2019; Wang et al., 2022). However, the direct effects of *Delftia* sp. on *C. vulgaris* have not been investigated. Compared to other *C. vulgaris*‐associated bacteria, *Delftia* sp. exhibits significant involvement in the nitrogen cycle, including atmospheric nitrogen fixation and reduction of ${\mathrm{NO}}_{3}^{-}$ to ${\mathrm{NO}}_{2}^{-}$, akin to *Microbacterium* sp. This resemblance is noteworthy, as *Microbacterium* sp. has been demonstrated to intensify the growth of *C. vulgaris*, particularly in environments where the preferred nitrogen source is ${\mathrm{NO}}_{3}^{-}$ rather than ${\mathrm{NO}}_{2}^{-}$ or ${\mathrm{NH}}_{4}^{+}$ (Kim et al., 2015). In addition, *Delftia* sp. has shown the ability to synthesize vitamin B12, as do lactic acid bacteria (LAB), which have been shown to positively affect carbon fixation and photosynthesis in *C. vulgaris* cells (Ji et al., 2021; Prabaningtyas et al., 2021; Ribeiro et al., 2023). These studies suggest that the presence of *Delftia* sp. in co‐cultivation scenarios may well contribute to the enhanced growth of *C. vulgaris* through various mechanisms, including nitrogen cycling and vitamin B12 synthesis.

In summary, our study highlights the critical importance of carefully selecting media components, especially the nutrient ratio (carbon and nitrogen) and microorganisms to establish efficient and cost‐effective species‐specific co‐cultures of algae and bacteria. By identifying potential algal–bacterial interactions, such as nutrient depletion and aggregate formation, responsible for the adverse effects of *Delftia* sp. in TAP media, proactive measures can be taken to minimize these effects during cultivation. This may involve optimization of bacterial inoculation strategies or supplementation of the growth medium with specific nutrients (carbon or nitrogen sources) or bacterial metabolites to enhance algal growth. Exploiting algal–bacterial interactions holds promise for achieving additional efficiencies, such as in wastewater treatment. The co‐cultivation of algae and bacteria can be used to improve the reduction of organic pollutants and heavy metals from wastewater, while producing valuable biomass for various industrial applications (Sepehri et al., 2020; Wang et al., 2022).

## CONCLUSION

Experiments and literature highlight two crucial factors shaping the interactions between algae and bacteria: the origin of the microorganisms with their different abilities to assimilate nutrients and the composition of the growth medium, which determine the availability of nutrients. In this study, a symbiotic interaction between *C. vulgaris* and *Delftia* sp. was initially proposed, which was ultimately found to be an adaptation to changing environmental conditions, with temporary microbial dominance depending on the growth conditions, where the composition of the growth medium is a key factor. Two phases of growth could be identified in the co‐culture systems that were studied: During the initial growth phase, *Delftia* sp. proliferate in all three media (TAP, BG‐11 and BG + AA), reaching peak densities within 2 d, indicating a high nutrient consumption, especially in the presence of organic carbon such as acetic acid (in TAP and BG + AA). In a second growth phase, 2–7 d, increased bacterial mortality was observed, peaking (>50%) at 6th d in BG‐11 and BG + AA. During this phase, *C. vulgaris* dominance prevailed, overcoming bacterial contamination and possibly benefiting from the nitrogen assimilation capabilities of the bacteria, including the release of ammonium, as well as possible contributions from phytohormones such as IAA (BG‐11 and BG + AA). However, when co‐cultured in TAP medium, the growth of *C. vulgaris* was slightly inhibited. Chemical analysis of the medium showed that the shifts in this interaction were influenced by the availability of organic carbon (acetic acid), which is consumed by both microorganisms. In all co‐cultured systems, we achieved stable algal cultures with steady or declining bacterial populations towards the cultivation's end, albeit not reaching the highest algal biomass yields observed in the absence of bacteria, as in the TAP medium. Our results highlight the importance of accurately assessing and adjusting nutrient ratios within growth media to effectively optimize co‐culture systems. However, further analytical methods such as metabolomics are essential to unravel the complex dynamics of nutrient composition, refine the understanding of interspecies interactions and facilitate the development of cost‐effective, environmentally friendly algal cultivation systems.

## AUTHOR CONTRIBUTIONS

**Kamile Jonynaite:** Conceptualization (equal); data curation (equal); investigation (lead); methodology (equal); validation (equal); visualization (equal); writing – original draft (equal). **Arunas Stirke:** Conceptualization (equal); methodology (equal); resources (equal); writing – review and editing (equal). **Henri Gerken:** Investigation (supporting); software (supporting); visualization (supporting); writing – review and editing (equal). **Wolfgang Frey:** Project administration (lead); resources (equal); writing – review and editing (equal). **Christian Gusbeth:** Conceptualization (equal); formal analysis (equal); investigation (equal); methodology (equal); supervision (lead); writing – original draft (equal).

## CONFLICT OF INTEREST STATEMENT

The authors of this article have no conflicts of interest to declare.

## Supporting information


**Data S1.** Supplementary information.

## Data Availability

Data supporting the findings of this study are available within the paper and the Supporting Information. Raw data files are available from the corresponding author upon reasonable request. The raw data of the 16S RNA gene sequence has been deposited into the GenBank (accession number OP777498).
