# Supplementary material for: Influence of growth medium on the species‐specific interactions between algae and bacteria
Source: Environ Microbiol Rep. 2024 Aug 21;16(4):e13321. doi: 10.1111/1758-2229.13321 (PMC11338630; doi:10.1111/1758-2229.13321)
Supplement: Supplementary file 1 — Data S1. Supplementary information. [file EMI4-16-e13321-s001.docx]

Article title: Investigating Species-Specific Algal/Bacterial Co-Culture Dynamics: Influence of Growth Medium

Authors: Kamile Jonynaite, Arunas Stirke, Henri Gerken, Wolfgang Frey, Christian Gusbeth

The following Supporting Information is available for this article:

**Fig. S1** Percentage of aggregated algae.

**Fig. S2** Delftia sp. bacterial growth in TAP or BG-11 medium.

**Fig. S3** Change in optical density, algae viability and pH of algae, bacteria and co-cultures in TAP medium.

**Fig. S4** Change in optical density, algae viability and pH of algae, bacteria and co-cultures in BG-11 medium.

**Fig. S5** Change in optical density, algae viability and pH of algae, bacteria and co-cultures in BG-AA medium.

**Fig. S6** Changes in NO_2_ concentration of co-cultures under TAP, BG-11 and BG+AA conditions.

**Fig. S7** Changes in NH_4_ concentration of co-cultures under BG-11 and BG+AA conditions.

**Table S1** Overview of species-specific algae-bacteria interactions that alter algal growth.

**Table S2** Biochemical properties of an unknown isolate and a comparison between D. lacustris 332T; D. tsuruhatensis NBRC 16741; D. acidovorans DSM.

**Fig. S1** Percentage of aggregated algae cultured in TAP, BG-11, and BG-AA media, with or without Delftia sp. bacteria.

**

**

**Fig. S2** Delftia sp. bacterial growth in TAP or BG-11 medium. Population dynamics assessed via optical density (A), CASY cell counter (B) and colony forming units (C).





**Fig. S3** Co-culture of C. vulgaris and Delftia sp. in TAP medium. Change in optical density (A), algae viability (B), (C). Significant differences (p<0.05) between data points within one day are marked with *.





**Fig. S4** Co-culture of C. vulgaris and Delftia sp. in BG-11 medium. Change in optical density (A), algae viability (B), pH (C). Significant differences (p<0.05) between data points within one day are marked with *.





**Fig. S5** Co-culture of C. vulgaris and Delftia sp. in BG+AA medium. Change in optical density (A), algae viability (B), pH (C). Significant differences (p<0.05) between data points within one day are marked with *.





**Fig. S6** Changes in NO_2_^-^ concentration in C. vulgaris and Delftia sp. grown together under TAP (A), BG-11 (B) and BG+AA (C) conditions. Significant differences (p<0.05) between data points within one day are marked with *.





**Fig. S7** Changes in NH_4_^+^ concentration in C. vulgaris and Delftia sp. grown together under BG-11 (A) and BG+AA (B) conditions. The dashed horizontal line indicates the initial concentration of the NH_4_^+^in the medium, calculated from the composition of the medium. Significant differences (p<0.05) between data points within one day are marked with *.





**Table S1** Examples of species-specific algae-bacteria interactions that alter algal growth. ND - not determined.

| Microalgae | Bacteria | Suggested mechanism | Ref. |
| --- | --- | --- | --- |
| *Chlorella vulgaris* and *Scenedesmus acutus* | *Flavobacterium, Hyphomonas, Rhizobium and Sphingomonas, Microbacterium* and *Exophiala* | Exchange of O_2_-CO_2_ and supplementation with essential nutrients | (Cho et al., 2015) |
| *Scenedesmus sp.* | *Pseudomonas sp., Stenotrophomonas sp., Achromobacter sp., Bacillus sp.* and *Acinetobacter sp.* | Synthesis and secretion of IAA | (Dao et al., 2018) |
| *Chlorella vulgaris* | *Rhizobium sp.* | ND | (Ferro et al., 2019) |
| *Chlorella sorokiniana* | *Melaminivora jejuensis* | Biofilm formation | (Kim et al., 2020) |
| *Scenedesmus vacuolatus* and *Haematococcus lacustris* | *Methylobacterium spp.* | Synthesis and secretion of vitamins, siderophores and plant hormones | (Krug et al., 2020b) |
| *Chlorella sorokiniana* and *Chlorella minutissima* | *Azospirillum brasilense, Escherichia coli* and *Bacillus megaterium* | Synthesis and secretion of auxin phytohormone indole-3-acetic acid (IAA), release of the riboflavin metabolite, lumichrome and O_2_-CO_2_ exchange | (Peng et al., 2021) |
| *Isochrysis galbana* | *Marinobacter sp.* | ND | (Wang, 2022) |

**Table S2** Biochemical properties of an unknown isolate and D. lacustris 332T; D. tsuruhatensis NBRC 16741; D. acidovorans DSM 39T and a comparison between them. Abbreviations: +, positive; −, negative; ND, not reported/not determined. Data were obtained in this study and from the literature.

|  | Isolate | *Delftia lacustris* | *Delftia tsuruhatensis* | *Delftia actidovorans* |
| --- | --- | --- | --- | --- |
| **ADH**  (Hydrolysis of arginine) | − | − | −/+ | − |
| **TRD**  (Utilization of thiosulfate) | + | ND | ND | + |
| **EST**  (Hydrolysis of lipid) | + | + | + | + |
| **PHS**  (Hydrolysis of phosphoester) | − | + | ND | + |
| **NAG**  (Hydrolysis of N-acetyl-β, D-glucosaminide) | − | + | − | − |
| **αGLU**  (Hydrolysis of α-glucoside) | − | + | − | − |
| **βGLU**  (Hydrolysis of β-glucoside) | − | + | − | − |
| **ONPG**  (Hydrolysis of β, D-galactoside) | − | ND | − | − |
| **URE**  (Hydrolysis of urea) | − | ND | + | − |
| **GLU**  (Utilization of D-glucose) | − | − | − | − |
| **PRO**  (Hydrolysis of Proline) | − | ND | ND | + |
| **PYR**  (Hydrolysis of Pyrrolidine) | − | ND | ND | + |
| **GGT**  (Hydrolysis of γ-Glutamyl) | + | ND | ND | + |
| **TRY**  (Hydrolysis of Tryptophan) | − | − | + | − |
| **BANA**  (Hydrolysis of N-Benzyl-arginine) | − | ND | ND | − |
| **IND**  (Formation of indole) | − | − | − | − |
| **NO3**  (Utilization of nitrate) | + | + | + | + |
